# Finding the gap: An empirical study of the most effective shots in elite goalball

**DOI:** 10.1371/journal.pone.0196679

**Published:** 2018-04-26

**Authors:** Daniel Link, Christoph Weber

**Affiliations:** Department of Exercise Science and Sport Informatics, Technical University Munich, Munich, Germany; Bern University of Applied Science, SWITZERLAND

## Abstract

This research identifies which shots types in goalball are most likely to lead to a goal and herby provides background information for improving training and competition. Therefore, we observed 117 elite level matches including 20,541 shots played in the regular situation (3 vs. 3) using notational analysis. We characterized the shots by using their target sector (A-E), technique (traditional, rotation), trajectory (flat, bounce), angle (straight, diagonal and outcome (goal, violation, out, blocked). In our data, a χ^2^-test showed a significantly higher goal rate for men (3.9%) compared to women (3.0%). For men, we found a significantly higher goal rate in the intersection sectors between players C (5.6%), D (4.9%), and in the outer sector A. In sector A, goal rate was higher only for straight shots (6.6%). Technique and trajectory did not affect goal rate for men, but flat shots showed a higher violation rate (3.2%) compared to bounce shouts (2.0%). In women's goalball, goal rate was higher only on sector D (4.4%). Bounce-rotation shots were the most successful (5.5%). We conclude that men should focus on shots to sectors C and D (called pocket) and straight shots to sector A, as long as there are no other tactical considerations. Women should shoot primarily towards the pocket. It might also be worth playing more bounce-rotation shots and practicing them in training.

## Introduction

**Fig 1:**
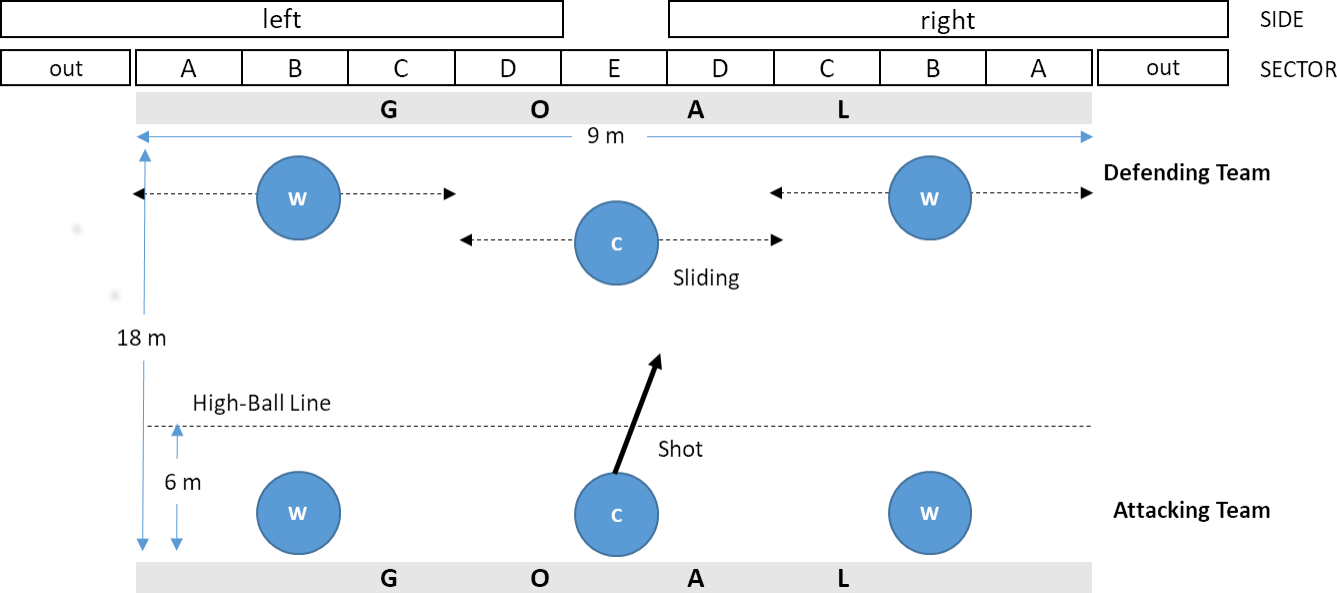
Geometry of goalball pitch, regular position of centre (c) and wing (w) players and operationalisations used.

Goalball is a Paralympic sport designed for visually impaired and blind people. The game consists of two teams of three players, played on a modified volleyball court with tactile markings allowing the players to determine their position [1]. The purpose of the game is to throw the ball from the one team’s area into the opposing team’s goal. The players on the defending team use their bodies to block the ball before it crosses their goal line. The ball contains bells allowing players to estimate its speed and direction. Once the defending team gain possession, it is their turn to attack. The ball must touch the ground in the area in front of the highball line (see Fig 1). If the ball touches the ground behind the highball line without touching the ground before it, the opponent team get a penalty, in which only one player is allowed to defend the ball. Players wear blindfolds to guarantee equal conditions.

Existing research covers different aspects of the game. One group of studies analysed physical fitness of visually impaired adolescents [2–5]. Psychologically oriented works concerned with mental imagery for top players [6] and the effects of self-talk during a penalty-situation [7]. A paper published by da Silva, Pereira, Depra, and Gorla [8] studied the correlation between reaction time and defence performance. Biomechanical research analysed different shot techniques and their impact on ball speed [9]. Other studies focussed on physical properties of the ball [10], physiological load during matches [11], anthropometric characteristics of top level players [12,13], requirement profiles for goalball coaches [14], and provided guidance for teaching in schools [15]. In addition, goalball-specific testing methods were developed for auditory perception [16] and endurance [17].

Compared to other team sports, the tactical structure of goalball is quite simple. One tactical decision a player has to make is which *sector* of the goal to play the ball into. In the regular situation (3 vs. 3), the defence line consists of three players, so that the target could be a defender, the gaps between the central defender and the outside defender—we refer to them as the *pocket—*as well as the space between an outside defender and a post (Fig 1). Depending on the starting position and the target sector, the ball hits the defence line at different *angles*. Regarding the shot’s *technique*, it is possible to distinguish between *traditional* shots and *rotation* shots. The movement of the traditional shot is similar to bowling while in rotation shots, players perform a 360° turn before releasing the ball [9]. A further shot parameter is the ball *trajectory*. In a *bounce* shot, players throw the ball to the ground at a steep angle, causing it to bounce several times on its way to the goal. In contrast, a *flat* shot shows a rather smooth trajectory of the ball.

Performance analysis needs to clarify whether the different shot types have different success probabilities. To date, the body of research in this area is quite limited. A poster presentation by Lehto et al. [18] studied goal rates of penalties and regular situations based on a sample of six men’s and six women’s elite level matches. A follow-up study based on a sample of 15 men’s matches revealed higher goal rates for bounce shots compared to flat shots, but without considering different target zones or throwing techniques [19]. An unpublished Master’s thesis by Owen [20] studied 44 elite women’s matches and reported that goals were scored primarily by attempts through the pocket. Furthermore, players scored more goals with flat shots than with bounce shots. However, without knowing the distribution of the shot types in this sample, it is not possible to draw a conclusion about success probabilities. Both studies did not distinguish between the regular and the penalty situation, which are completely different [7]. Further, there is a paper [21] as well as an unpublished dissertation [22] in Portuguese. Morato et al. [23] described an observation scheme for goalball, but without presenting empirical data. To our knowledge, no comprehensive study investigating the tactical structure of goalball exists.

This study examines the distribution and goal rate of different shot types in men’s and women’s elite goalball using notational analysis [24]. We aim to answer the question of which shot types are most likely to lead to a goal. Characterisation of shots uses variables which have–in our opinion–a potential impact on success. These are: the target sector, the angle at which the ball hits the defence line, the throwing technique, and the ball trajectory. Our analysis is limited to the regular situation, in which more than 96% of the attempts take place [18]. The results are potentially useful for the development of match strategies and the education of coaches and players.

## Methods

### Sample

In line with the objectives of our study, we applied a non-participative observational approach. The sample comprises 113 matches (63 men, 50 women) of 2012 and 2016 Paralympic competitions and 2013 and 2015 European Championships. Since each player agreed to the video recording of matches on signing their player license, special approval for this study from an ethics committee was not required. Nevertheless, all procedures performed in the study were in strict accordance with the declaration of Helsinki as well as with the ethical standards of the local ethics committee.

### Performance variables

For each shot played in the regular situation, we collected performance parameters according to Table 1 and Fig 1. Shots played with very low speed solely in order to consume more time, were excluded from the analysis. All data was annotated by a professional goalball analyst, who was a part of the German national goalball team, by using custom made observation software [25]. Observations based on self-made video recordings, which were taking by a single camera located behind the court. Annotations and video recordings were also used for preparing Germany’s team for their competitions.

**Table 1:** Used performance variables, categories and operationalisations. In the text, we refer to the type by combing its ANGLE, TRAJECTORY and TECHNIQUE. For example, a shot played with a bouncing trajectory and the rotation technique is referred bounce-rotation shot. If only one variable is important, we call it e.g. diagonal shot.

Variable	Categories
**GENDER**	*men* *women*
**SECTOR**	*[A*, *B*, *C*, *D*, *E*, *Out]*: Segment, where the ball trajectory intersects the goal line or the predicted intersection point, when a player blocks the ball before (Fig 1).
**SECTOR’**	*[A*, *B*, *C*, *D*, *E]*: Segment similar to SECTOR. A contains additional all Outs. In E, each shot is counted twice. B, C, D are equal to the categories of SECTOR.
**SIDE**	*[left*, *right]*: *4m* Segment according to Fig 1.
**ANGLE**	*straight*: Angle between the ball trajectory and the goal line is between 75 and 90°. *diagonal*: Otherwise
**TECHNIQUE**	*rotation*: Before releasing the ball, the player performs a 360-degree rotation around his longitudinal axis. *traditional*: Otherwise. Player performs a movement that is similar to bowling.
**TRAJECTORY**	*bounce*: The player throws the ball on the ground at a steep angle, causing it to bounce several times on its way to the goal *flat*: Otherwise. The player throws the ball on the ground in a sharp angle. The intension is to reach an almost smooth and fast trajectory.
**OUTCOME**	*goal*: The referee decided on goal for the attacking team. *out*: The referee decided on Out. It is not relevant if a player touches the ball before. *violation*: The referee decided on penalty for the defending team. *block*: Otherwise.

SECTOR and ANGLE were calculated based on the ball trajectory in the video and a calibration of the pitch. Cohen's kappa statistics showed substantial to perfect inter-rater agreement between two observers based on a subset of 548 shots in 3 matches (κ_GENDER_ = 1.0, κ_SECTOR_ = .94, κ_SIDE_ = .99, κ_ANGLE_ = .96, κ_TECHNIQUE_ = 1.0, κ_TRAJECTORY_ = .84, κ_OUTCOME,_ = .98). For testing intra-rater agreement, these 3 matches were observed two times by the same goalball analyst with a time gap of 6 month. Kappa statistics show very similar results (κ_GENDER_ = 1.0, κ_SECTOR_ = .96, κ_SIDE_ = .99, κ_ANGLE_ = .97, κ_TECHNIQUE_ = 1.0, κ_TRAJECTORY_ = .95, κ_OUTCOME,_ = .98).

### Statistical analysis

On shot level we report the results in the categorical variables GENDER, SECTOR, SECTOR’, SIDE, TECHNIQUE, TRAJECTORY and ANGLE. We also studied interaction effects between TECHNIQUE × TRAJECTORY, TRAJECTORY × ANGLE, SECTOR’ × ANGLE and SECTOR’ × ANGLE × TRAJECTORY. For each category, we calculated the incidence rate (#), as the number of shots in a category divided by the sum of all shots, and also the goal rate (GR), violation rate (VR), and the out rate (OR) as the number of these incidents divided by the number of shots in this category. When comparing two rates between conditions, we report their difference (Δ) in signed (+,-) percentages of the reference value. On match level, data is presented as the mean ± standard deviation.

To test the differences between ratios we applied a χ^2^-post-hoc test, which compares each cell with the mean of all others. Cramer’s v is used to describe the effects size of significant differences. To study interaction effects between conditions, we used χ^2^-test followed by Marascuilo’s procedure, which determines pairwise differences between cells. The multifactorial analysis of SECTOR’ considers sector A’ and the pocket sector CD (which contains all shots in sectors C and D). Before using parametric statistical test procedures, we verified the assumption of normality. The alpha level was set to .05. All statistical analyses were performed using SPSS Statistics 23 (IBM Corp., USA).

## Results

In our sample, there were 20,541 shots in total (11,451 for men and 9,090 for women). The number of shots per match was 184.2 ± 20.6 for men and 181.3 ± 20.9 for women. There were significant differences between men and women regarding OUTCOME (χ^2^ = 65.8, p < .001, v = .06). In men matches, the goal rate was +30.0% higher than in women’s matches (Table 2). Violation rate was +116.6% higher for men (men: VR = 2.6%; women: VR = 1.2%). Per match, men scored more goals (9.9 ± 5.0) and more penalties (3.6 ± 3.6) than women (6.8 ± 4.1 goals; 2.7 ± 3.3 penalties). Overall, men scored 72.0% and women 79.8% of goals in in the regular situation.

**Table 2:** Incidences rate (#) and goal rates (GR) in goalball’s regular situation grouped by GENDER, SIDE, SECTOR, SECTOR’, TECHNIQUE, TRAJECTORY and ANGLE.

	men		women	
	#	GR	#	GR
Total	100	3.9	100	3
SECTOR				
Out	10.9*^<^	-	9.7*^<^	-
A	14.1*^<^	5.8*^>^	15.6*^<^	3.3
B	19.9*^>^	2.8*^<^	20.9*^>^	2.6
C	22.9*^>^	5.6*^>^	21.0*^>^	4.4*^>^
D	21.4*^>^	4.9	22.3*^>^	3.5
E	10.9*^<^	3.1*^<^	10.6*^<^	2.0*^<^
SECTOR’				
A	22.5*^>^	3.3*^<^	22.8*^>^	2
B	17.9*^<^	2.8*^<^	18.9*^<^	2.6
C	20.6	5.6*^>^	19	4.4*^>^
D	19.3	4.9*^>^	20.1	3.5*
E	19.6	3.1*^<^	19.1	2.0*^<^
SIDE				
left	44.8	4.1	49.5	2.8
right	45.7	3.8	50.5	3.1
ANGLE				
straight	69.5*^>^	4.3*^>^	68.6*^>^	3.5*^>^
diagonal	30.5*^<^	3.2*^<^	31.4*^<^	2.0*^<^
TRAJECTORY				
flat	37.4*^<^	3.6	73.5*^>^	2.4*^<^
bounce	62.6*^>^	4.2	26.5*^<^	4.6*^>^
TECHNIQUE				
traditional	40.6*^<^	3.8	75.2*^>^	2.5*^<^
rotation	59.4*^>^	4	24.8*^<^	4.4*^>^

* indicates significant differences between cells.

^< >^ indicates, if value is greater respectively less than the group mean value.

Incidence rate differed in men matches according to SECTOR, with the sectors B, C, and D being above the mean value and sector A, E and Out being significantly below (Δ_B_ = +19.4%, Δ_C_ = +37.4%, Δ_D_ = +28.4%, Δ_A_ = -11.1%, Δ_E_ = -34.6%, Δ_Out_ = -34.6%, χ^2^ = 515.7, p < .001, v = .15). A similar characteristic was apparent in women matches (Δ_B_ = +25.4%, Δ_C_ = +26.0%, Δ_D_ = +33.8%, Δ_A_ = -6.4%, Δ_E_ = -36.4%, Δ_out_ = -41.8%, χ^2^ = 335.0, p < .001, v = .14). There were also differences between men and women (χ^2^ = 26.3, p < .001, v = .04). Male players hit sector C by +9.0% and sector Out by +12.4% significantly more frequently and sector A by -9.6% less frequently than female players. In men’s matches, goal rate was increased by +48.7% in sector A, by +43.6% in sector C and in women’s matches in sector C by +46.7% compared to the mean goal rate (men: χ^2^ = 45.3, p < .001, v = .07; women: χ^2^ = 15.3, p < .01, v = .04). A significantly lower goal rate was evident in sector E for both men by -20.5% and women by -33.3%. According to SIDE, neither incidence rate nor goal rate differed between men and women.

Analysis of SECTOR’ indicates a higher incidence rate in sector A, and a lower incidence rate in sector B compared to the mean incidence rate per sector (men: Δ_A_ = +12.5%, Δ_B_ = -10.5%, χ^2^ = 16.7, p < .01, v = .04; women: Δ_A_ = +14.0, Δ_B_ = -5.5%, χ^2^ = 29.8, p < .001, v = .04). There were only differences in incidence rates between men and women in sector C, which was somewhat more commonly played by men (Δ = +8.1%, χ^2^ = 12.7, p < .05, v = .03). In men’s matches, goal rate was higher in sector C by +43.6% and D by +25.6% and lower in sector A by -0.6%, B by -15.4% and E by -20.5% (χ^2^ = 4.3, p < .05, v = .02) compared to the mean goal rate. For women’s matches goal rate was only higher in sector C by +46.7% and lower in sector A by-33.3% and E by-33.3% (χ^2^ = 15.3, p < .01, v = .04).

ANGLE shows a higher incidence rate of straight shots compared to diagonal shots (men: Δ = +127.9%, χ^2^ = 907.2, p < .001, v = .20; women: Δ = +118.5%, χ^2^ = 649.8, p < .001, v = .19). Straight shots were also more successful than diagonal shots for men and women (men: Δ = +25.6%, χ^2^ = 8.4, p < .01, v = .03; women: Δ = +42.9%, χ^2^ = 15.3, p < .01, v = .04). Out rate was lower for straight shots compared to diagonal shots (men: OR_straight_ = 7.1%, OR_diagonal_ = 20.0%, Δ = -64.5%, χ^2^ = 407.9, p < .001, v = .19; women: OR_straight_ = 8.2%, OR_diagonal_ = 16.2%, Δ = -49.4%, χ^2^ = 130.9, p < .001, v = .12). If the out shots are excluded from the sample, the difference in goal rate between diagonal and straight shots was significant for women (GR_straight_ = 3.8%, GR_diagonal_ = 2.4%, Δ = +58.3%, χ^2^ = 10.1, p < .01, v = .04) but not for men (GR_straight_ = 4.8%, GR_diagonal_ = 4.0%).

Men and women differed in incidence rate according to TRAJECTORY (χ^2^ = 2649.6, p < .001, v = .36). While men’s teams played +67.4% more bounce shots than flat shots, women played +177.4% more flat shots. The goal rate did not differ between bounce shots and flat shots in men’s matches, whereas in women’s matches bounce shots were by +91.7% more successful than flat shots (χ^2^ = 28.0, p < .01, v = .06). Violation rate was higher for flat shots than for bounce shots for men and women (men: VR_flat_ = 3.2%, VR_bounce_ = 2.0%, Δ = +60.0%, χ^2^ = 24.6, p < .001, v = .05; women: VR_flat_ = 0.8%, VR_bounce_ = 1.5%, Δ = +87.5%, χ^2^ = 5.1, p < .05, v = .02).

Men and women also showed different incidence rate in the categories of TECHNIQUE (χ^2^ = 2458.1, p < .001, v = .35). Men played +46.3% more rotation shots than traditional shots, whereas women played +203.2% more traditional shots. In women’s matches, goal rate of rotation shots was +76.0% higher (χ^2^ = 19.0, p < .01, v = .05) and out rate was -26.1% lower compared to traditional shots (GR_traditional_ = 11.5%, GR_rotation_ = 8.5%, χ^2^ = 15.8, p < .001, v = .04). In men’s matches, there was no significant difference between techniques in goal rate or out rate. Similarly, there was no difference between women or men in violation rate between traditional shots and rotation shots. Analysis of TRAJECTORY x TECHNIQUE shows that bounce shots were more often combined with the rotation technique and flat shots with the traditional technique (χ^2^ = 1397.8, p < .001, v = .16) (Table 3). In women’s matches, bounce shots were only more successful than straight shots when played with the rotation technique (Δ = +120.0%, χ^2^ = 35.4, p < .001, v = .06).

**Table 3:** Incidence rates (#) and goal rates (GR) in goalball’s regular situation grouped by GENDER, TRAJECTORY × TECHNIQUE, TRAJECTORY × ANGLE, SECTOR’ × ANGLE and SECTOR’ × ANGLE × TRAJECTORY.

	men		women	
	#	GR	#	GR
TRAJECTORY × TECHNIQUE				
flat-traditional ^a^	20.5^bcd^	3.9	66.0^bcd^	2,5^d^
flat-rotation ^b^	17.0^acd^	3.4	7.6^acd^	2.5^d^
bounce-traditional ^c^	20.1^abd^	4	9.2^abd^	3,6
bounce-totation ^d^	24.4^abc^	4.5	17.2^abc^	5,5^ab^
TRAJECTORY × ANGLE				
straight-flat ^a^	26.7^bcd^	4.3^c^	48.7^bcd^	3.0^bc^
straight-bounce ^b^	42.8^acd^	4.6^c^	19.9^acd^	5.2^ac^
diagonal-flat ^c^	10.7^abd^	2.3^ab^	24.8^abd^	1.6^ab^
diagonal-bounce ^d^	19.8^abc^	3.8	6.6^abc^	3.6
SECTOR’ × ANGLE				
A straight ^a^	18.2^bc^	6.6^b^	32.9^bcd^	2.8^bc^
A diagonal ^b^	22.6^acd^	1.0^acd^	13.0^acd^	1.0^ac^
CD straight ^c^	40.0^abd^	5.7^b^	45.0^abd^	4.7^abd^
CD diagonal ^d^	19.3^bc^	5.0^b^	18.1^abc^	2.6^c^
SECTOR’ × ANGLE × TRACETORY				
A straight-flat ^a^	19.3^bcd^	7.4^cd^	50.5^bcd^	2.4^c^
A straight-bounce ^b^	25.3^ad^	5.8^cd^	14.2^acd^	4.5^c^
A diagonal-flat ^c^	26.3^a^	1.1^ab^	28.2^abd^	0.6^ab^
A diagonal-bounce ^d^	29.0^ab^	1.0^ab^	7.1^abc^	2.5
CD straight-flat ^a^	25.9^bcd^	5.4	47.9^bcd^	3.8
CD straight-bounce ^b^	47.4^acd^	5.9	23.4^ad^	6.5^c^
CD diagonal-flat ^c^	8.4^abd^	3.3	22.4^ad^	2.2^b^
CD diagonal-bounce ^d^	18.3^abc^	5.7	6.3^abc^	4.2

^abcd^ indicate significant differences to other cells.

Significant TRAJECTORY x ANGLE interactions were found for involvement with goal rate. Success of straight shots was higher than for diagonal-flat shots (men: Δ = +87.0%, χ^2^ = 11.4, p < .01, v = .04; women: Δ = +118.8%, χ^2^ = 4.5, p < .05, v = .03). In contrast, goal rate did not significantly differ from straight shots to diagonal-bounce shots. If out shots are excluded, the interaction effect persists only in women matches (Δ = +111.1%, χ^2^ = 30.4, p < .001, v = .06).

Interaction effects on SECTOR x ANGLE occurred only in men’s matches. In sector A, +24.2% more diagonal shots than straight shots were played, in sector DC there were –51.5% less diagonal shots than straight shots (χ^2^ = 1155.3, p < .001, v = .18). In sector A, goal rate of diagonal shots was lower in comparison to straight shots (Δ = -84.8%, χ^2^ = 56.2, p < .001, v = .14), whereas in sector CD there are no significant differences between diagonal shots and straight shots. Diagonal shots in sector A showed a distinctly higher out rate compared to straight shots (Δ = +363.7%, χ^2^ = 52.2, p < .001, v = .12).

Analysis of SECTOR x ANGLE x TRAJECTORY showed no significant interaction effects. Among all combinations, in men’s matches, straight-flat shots to sector A showed the highest goal rate, which is +89.7% higher compared to the mean (χ^2^ = 13.2, p < .001, v = .03). The second highest goal rate was in sector CD for straight-bounce shots (Δ = +51.3%, χ^2^ = 32.0, p < .001, v = .04). For women’s matches the highest goal rate occurred in sector CD for straight-bounce shots. Compared to mean goal rate, this is an increase of 116.7% (χ^2^ = 24.9, p < .001, v = .04). In sector A, straight-bounce shots were by +50.0% the most successful, but goal rate was not significantly different from the mean.

## Discussion

This study aimed to analyse the occurrence and the success of different shot types in elite goalball in order to identify the most promising variants. Compared to Owen [20] and Morato [22,23], a finer classification (9 not 7) of target zones was used. Symmetrical areas on the left and right hand side of the courts’ longitudinal axis were joined to one sector, since no laterality effects occurred in the data and the statistical validity could be increased in this way. For the same reason we joined sectors C and D when analysing shots to the pocket in detail. SECTOR’ is used to represent incidence rate in relation to the intended target sector. To achieve this, out shots were assigned to sector A (because the players usually do not throw the ball out deliberately) and the shots to sector E were counted twice (to compensate for the half width of the sector). SECTOR’ is also used to analyse goal rate in consideration of out shots.

The number of shots, goals and violations per match were similar to the results reported by Letho et al. [18]. The higher goal rate for men can be attributed to their greater physical strength [26], leading to higher ball speeds and higher bouncing amplitudes compared to womens’ goalball. This suggests that advantages in strength in goalball are more relevant in offense—characterized by powerful movements—than in defence, in which players attempt to produce a large passive target area. Both, the higher out rate and violation rate suggest a tactical decision to take more risks in men’s goalball.

According to SECTOR’, the distribution of shots was almost equally distributed for men and women—only sector A was played slightly more often. Shots to the pocket, represented by sector C and D, were most successful for both genders. This result is in line with Owen [20] and is justified by the fact that these sectors represent the gaps between the defending players in their starting position. In the defence, players dive laterally for the ball and therefore only hands and feet cover the gaps. The probability that a ball deflects off the defender’s extremities is higher, compared to the torso [18]. The same reasoning explains the higher goal rate in sector A in men’s matches. Additionally, there is also only one player who has the chance to block the ball.

For men matches, our data gives no indication of an influence of technique on goal rate. The rotation technique may have the potential to favour higher ball speeds due to the longer acceleration path [9], however, this advantage–if it exists–was too weak to influence goal rate significantly. Similarly, there is no general advantage of a particular ball trajectory. One could argue that the additional vertical movement in bounce shots increases the probability of the ball deflecting off the defender towards the goal. On the other hand, the speed of the ball compared to flat shots should be reduced by the steeper angle and the harder impact on the ground, thus reducing goal rate. Our data suggests that these counteracting effects in men’s matches either cancel each other out or do not exist.

In women’s matches, on the other hand, bounce-rotation shots were more successful. This finding is in line with Owen’s results [20], who reported a weak positive correlation between the team skill level and the percentage of bounce-rotation shots. Our data also suggests that the trajectory has a slightly higher influence on goal rate than the technique. The reason for this difference between the genders is not clear. It is striking that these types of shots were used much less frequently in women’s than in men’s matches. Possibly, to be successful, this variant requires better technique or higher strength. Therefore, female athletes that are stronger and/or more skilful—and have higher goal rate in general–might play these types of shots more often. However, this is somewhat speculative.

Regarding rotation shots, one might assume that the 360° rotation places makes orientation harder and leads to a lower precision and consequently more out shots. However, the data does not show this either in men’s or women’s matches. On the contrary, the out rate was even slightly lower with this technique in women’s matches. The higher violation rate in flat shots can be explained by the flatter angle when the ball strikes the ground. Here, the probability that the ball makes its first contact after the highball line is higher than for bounce shots. Considering that the goal rate from penalties is around 45% [18], this could be an additional argument for performing bounce shots.

Furthermore, diagonal-flat shots showed a lower goal rate in women’s matches–even if they hit the defenders and did not miss the goal. One reason could be a combination of lower speed through their longer and flatter trajectory, which makes defence easier. Our data suggests the same tendency for men’s matches, but there is no statistical proof. This could be either because diagonal-flat shots are still sufficiently fast to be dangerous, or men use this shot type less frequently, which influences the statistical test.

In men’s goalball, the deep analysis of sector A shows a higher goal rate, but in terms of SECTOR’, goal rate was below the mean. From a methodological standpoint, this can be attributed to the high out rate of diagonal shots, since these shots were assigned to sector A by definition. A second reason could be that a diagonal trajectory favours out shots. Since goal rate of straight shots on sector A was comparable to sector CD, this shot type was an effective tactical option for players. We also assume that these shots were particularly successful when they were played flat. Although, we could not prove this statistically, one could argue that straight-flat shots pass defenders more frequently because of the higher ball speed. Goal rate of shots to sector CD were generally above the mean. Here, we found no advantage for one specific shot type.

In women matches, goal rate in sector A did not differ from the mean and was even below the mean, if out shots were included. In contrast to men, straight shots to sector A did not show a higher goal rate. We assume that the lower ball speed reduces the probability of playing the ball past the defender for women. In this context, it is noteworthy that a comparatively high number of shots were played to sector A, which is not advisable in the light of the goal rate. Owen [20] also reported a weakly negative correlation between the success of a team and the proportion of throws to the edge. There are two interpretations: either less successful teams estimated the success rate of these shots incorrectly, or these shots were less successful. Finally, the existing data cannot clarify the causality. Shots to sectors CD in women’s matches were exceptionally successful, as in men’s goalball. To explain the higher goal rate of straight shots compared to diagonal shots, one could argue once again that the ball trajectory and consequently the time to react is shorter. We also suspect that straight-bounce shots to the pocket are the most promising ones for women, since they have the additional possibility of bouncing over the defenders. However, this could not be statistically proven with the data available.

In addition to the parameters used in this study, there some other variables which potentially influence success. For example, there are different options for arranging the defence line, depending on whether the central defender is before or behind the wing defenders [23]. With respect to ball trajectory, Letho et al. [19] used the additional category “curved”. This probably describes flat throws with a sidespin, which have a slightly curved trajectory. However, we considered the distinction from straight shots as difficult and did not use this category. Further parameters could be the defence strategy of the pocket (foot-hand, hand-hand, foot-foot), positional changes before the throw, temporal throwing sequence, and ball speed [20]. However, we think that the examined parameters covered the most important factors and thus provided an empirical basis for preferring particular shot tactics in goalball. For practical use, it is important to note that the data represent a large sample of shots of different teams in several matches. To be successful, each single tactical decision must consider factors such as strengths and weaknesses of individual players, options for counter attacks, variability, or the element of surprise.

## Conclusion

As long as there are no other tactical considerations, it is advisable in men’s goalball to play the ball either to the pockets or next to the goalpost. Shots to the goalpost should be almost straight; otherwise, the probability of missing the goal is disproportionately high. There is no empirical evidence for using a certain technique or trajectory for men. Women should shoot primarily towards the pockets, since the edge of the goal is less promising for all shot types. Bounce-rotation shots promise a higher goal rate in women’s goalball and it might be worthwhile practising them. Since goal rates of diagonal-flat shots are comparatively low, players should use them rarely—especially females.

## Supporting information

S1 File Annotation file of goalball matches. (CSV)
